# Immobilization of silver nanoparticles on polyethylene terephthalate

**DOI:** 10.1186/1556-276X-9-305

**Published:** 2014-06-16

**Authors:** Alena Reznickova, Zdenka Novotna, Zdenka Kolska, Vaclav Svorcik

**Affiliations:** 1 Department of Solid State Engineering, Institute of Chemical Technology Prague, Prague 6 166 28, Czech Republic; 2 Faculty of Science, J.E. Purkyne University, Usti nad Labem 400 96, Czech Republic

**Keywords:** Polymer, Plasma activation, Surface properties, Silver nanoparticle grafting, Atomic force microscopy (AFM), Transmission electron microscopy (TEM)

## Abstract

Two different procedures of grafting with silver nanoparticles (AgNP) of polyethylene terephthalate (PET), activated by plasma treatment, are studied. In the first procedure, the PET foil was grafted with biphenyl-4,4′-dithiol and subsequently with silver nanoparticles. In the second one, the PET foil was grafted with silver nanoparticles previously coated with the same dithiol. X-ray photoelectron spectroscopy and electrokinetic analysis were used for characterization of the polymer surface at different modification steps. Silver nanoparticles were characterized by ultraviolet-visible spectroscopy and by transmission electron microscopy (TEM). The first procedure was found to be more effective. It was proved that the dithiol was chemically bonded to the surface of the plasma-activated PET and that it mediates subsequent grafting of the silver nanoparticles. AgNP previously coated by dithiol bonded to the PET surface much less.

## Background

Immobilization of microspheres and nanoparticles (NPs) onto the surface of organic polymers provides fascinating opportunities for the design of smart heterostructures [1]. In addition to size, shape, and size uniformity, control of dispersion of NPs is a key parameter to minimize the loss of properties related to the nanosize regime [2].

Silver nanoparticles (AgNPs or nanosilver) have attracted increasing interest due to their unique physical, chemical, and biological properties compared to their macroscaled counterparts [3]. AgNPs have distinctive physicochemical properties, including a high electrical and thermal conductivity, surface-enhanced Raman scattering, chemical stability, catalytic activity, and nonlinear optical behavior [4]. These properties make them of potential value in inks, microelectronics, and medical imaging [5]. Besides, AgNPs exhibit broad-spectrum bactericidal and fungicidal activity [6] that has made them extremely popular in a diverse range of consumer products, including plastics, soaps, pastes, food, and textiles, increasing their market value [7]. To date, nanosilver technologies have appeared in a variety of manufacturing processes and end products. Nanosilver can be used in a liquid form, such as a colloid (coating and spray) or contained within a shampoo (liquid), and can also appear embedded in a solid such as a polymer master batch or be suspended in a bar of soap (solid). Nanosilver can also be either utilized in the textile industry by incorporating it into the fiber (spun) or employed in filtration membranes of water purification systems. In many of these applications, the technological idea is to store silver ions and incorporate a time-release mechanism. This usually involves some form of moisture layer that the silver ions are transported through to create a long-term protective barrier against bacterial/fungal pathogens [7,8].

**Figure 1:**
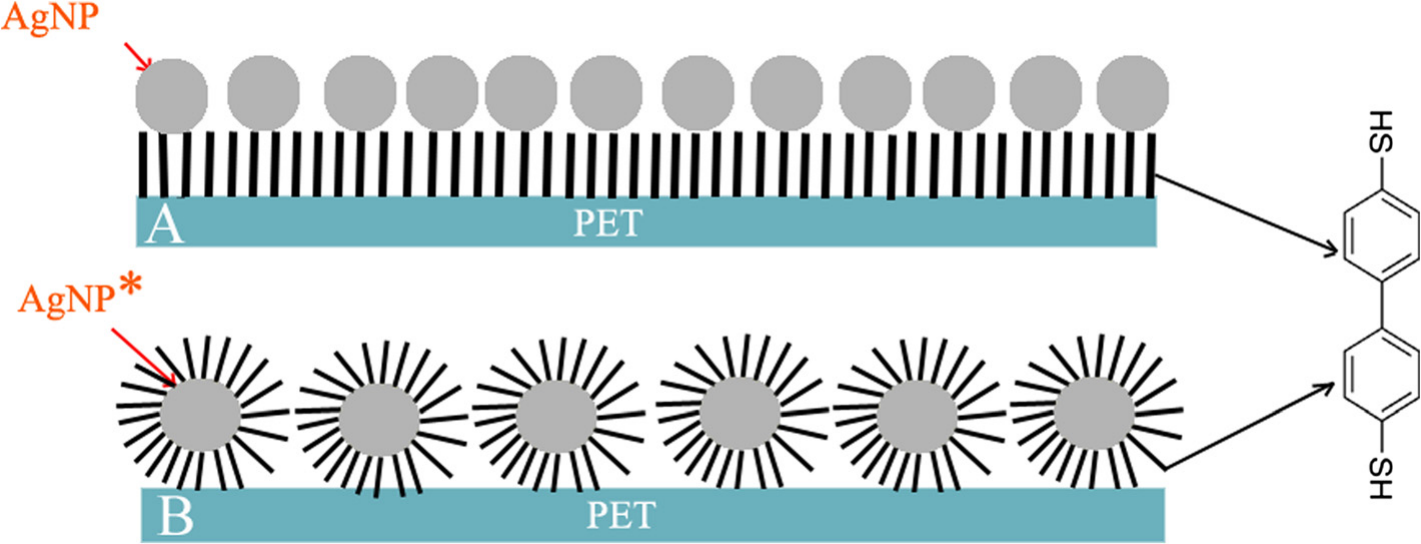
**Scheme of PET modification. (A)** Plasma treatment, grafting with dithiol (-SH) and then with silver nanoparticles (AgNP). **(B)** Plasma treatment, grafting with silver nanoparticles in advance coated with dithiol (AgNP*).

The chemical composition of the support is also important as virtually the number of polymeric platforms is unlimited, ranging from natural to synthetic ones. Homopolymers, copolymers, and block polymers can be synthesized from several monomers and monomer mixtures of different natures. In addition, polymer chain length and numerous combinations of monomers constituting the polymeric supports could be tuned in order to optimize the final polymeric material architecture and its performances. Another reason for the rush in designing polymeric platforms for anchoring nanoparticles is the ease of preparation via well-established chemical [9], electrochemical [10], and radiation-induced routes [2,11,12].The aim of this work was immobilization of AgNPs on a flexible substrate (polyethylene terephthalate (PET)). Such nanostructured surface could find application in, e.g., medicine as a surface with antimicrobial properties. Antibacterial behavior is of interest of our future studies. Two slightly different techniques were used for coating of PET surface with AgNPs. In the first procedure (A), the AgNPs were deposited on PET, beforehand grafted with biphenyl-4,4′-dithiol (BPD), and (B) in the second one, the silver nanoparticles (AgNP*), first coated with BPD, were deposited-grafted onto the plasma-treated PET (see Figure 1).

## Methods

### Materials and modification

Biaxially oriented polyethylene terephthalate (PET, density 1.3 g cm^-3^, 23-μm foil, supplied by Goodfellow Ltd., Huntingdon, UK) was used in this study. The samples were treated in Ar^+^ plasma on a Balzers SCD 050 device: the exposure time was 120 s, and the discharge power was 8.3 W. The plasma treatment was accomplished at room temperature. More detailed description of the plasma modification can be found in [13].

Immediately after the plasma treatment, the samples were inserted into a methanol solution of biphenyl-4,4′-dithiol (BPD, 4.10^-3^ mol l^-1^). Silver nanoparticles (AgNPs) were obtained using a similar process of AgNO_3_ reduction to that reported by Smith et al. [14]. Thiols are expected to be fixed via one of their functional -SH group to reactive sites created by the plasma-activated polymer surface [15]. The remaining ‘free’ -SH group is then allowed to interact with AgNPs [16]. Coating of polymers with AgNP*s was accomplished by two procedures (illustrated in Figure 1): (A) the plasma-treated polymer, grafted with BPD, was immersed into freshly prepared solution of silver nanoparticles (in what follows denoted as AgNP); and (B) the plasma-treated polymer was exposed to a solution of silver nanoparticles, previously coated with BPD (AgNP*) for 24 h. Finally, the samples were immersed into distilled water and then dried under N_2_ flow.

### Measurement techniques

For characterization of silver nanoparticles, transmission electron microscopy (TEM) images of silver nanoparticles (AgNP and AgNP*) were obtained on a JEOL JEM-1010 (JEOL Ltd., Tokyo, Japan) instrument operated at 80 kV. UV-vis absorption spectra of nanoparticles were recorded using a Varian Cary 400 SCAN UV-vis spectrophotometer (PerkinElmer Inc., Waltham, MA, USA). The solutions were kept in 1-cm quartz cell. Reference spectrum of the solvent (water) was subtracted from all spectra. Data were collected in the wave region from 350 to 800 nm with 1-nm data step at the scan rate of 240 nm min^-1^.

Different techniques were used for characterization of the modified polymer surface. Concentrations of C(1s), O(1s), S(2p), and Ag(3d) atoms in the modified surface layer were measured by X-ray photoelectron spectroscopy (XPS). An Omicron Nanotechnology ESCAProbe P spectrometer (Omicron Nanotechnology GmbH, Taunusstein, Germany) was used to measure photoelectron spectra (typical error of 10%).

Electrokinetic analysis (zeta potential) of all samples was accomplished on SurPASS Instrument (Anton Paar GmbH, Graz, Austria) to identify changes in surface chemistry and polarity before and after individual modification steps. Samples were studied inside the adjustable gap cell with an electrolyte of 0.001 mol l^-1^ KCl, and all samples were measured eight times at constant pH = 6.0 and room temperature (error of 5%). Two methods, streaming current and streaming potential, were used to evaluate measured data, and two equations, Helmholtz-Smoluchowski (HS) and Fairbrother-Mastins (FM), were used to calculate zeta potential [17].

Surface morphology was examined by atomic force microscopy (AFM) using a Veeco CP II setup (tapping mode) (Bruker Corporation, Billerica, MA, USA). Si probe RTESPA-CP with a spring constant of 0.9 N m^-1^ was used. By repeated measurements of the same region (2 × 2 μm^2^ in area), we proved that the surface morphology did not change after five consecutive scans.

## Results and discussion

**Figure 2:**
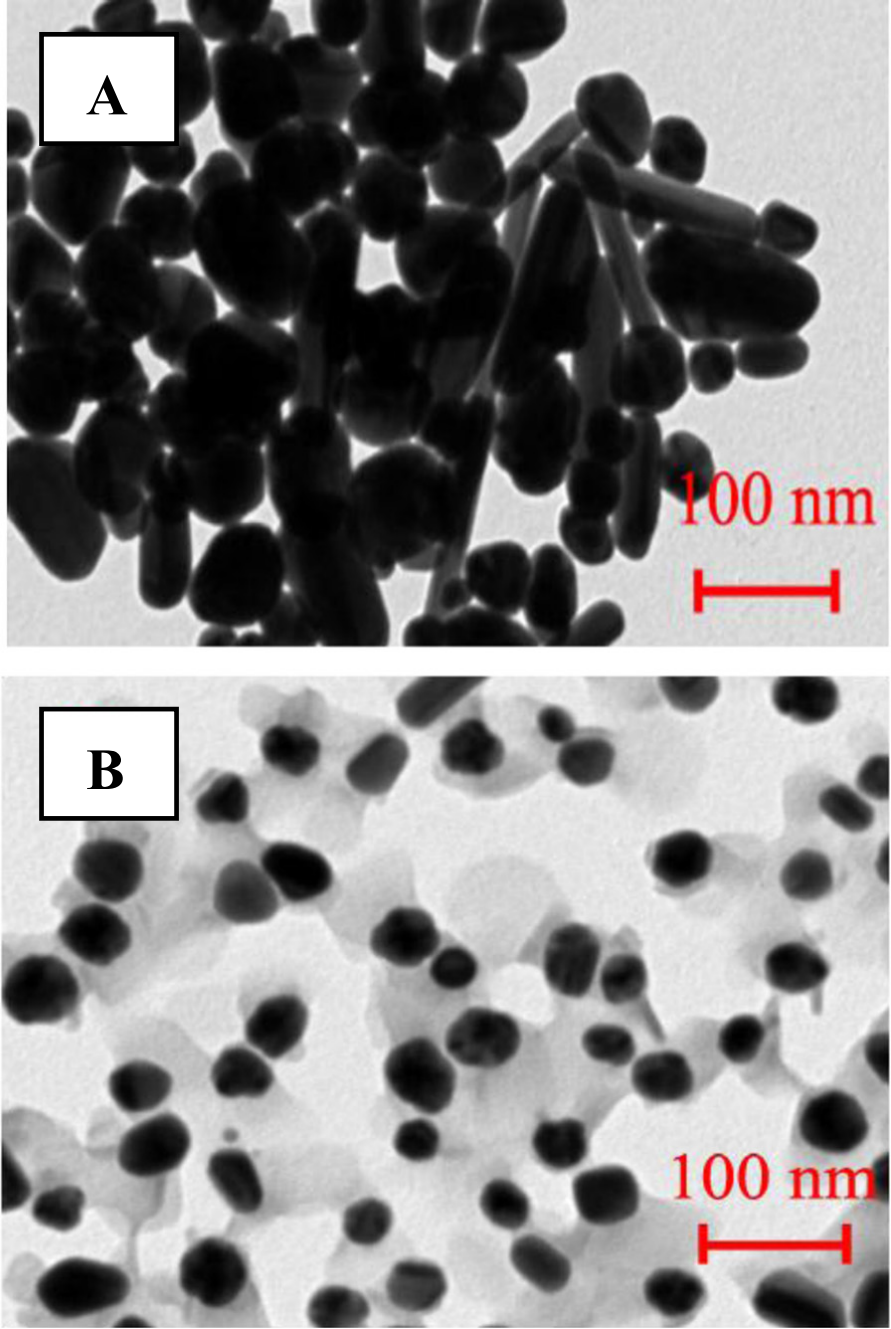
TEM images of silver nanoparticles (A, AgNP) and silver nanoparticles coated with dithiol (B, AgNP*).

Two procedures of immobilization of AgNPs on the surface of PET are illustrated in Figure 1. The prepared structures were first examined by TEM (Figure 2A, B). It is seen that the behavior of naked AgNPs (AgNP-2A) and AgNPs coated by BPD (AgNP*-2B) is dramatically different. While AgNPs create quite uniform aggregates of nonspherical shape, AgNPs* have spherical shape and they are well dispersed. Grafting with BPD does not lead to AgNP aggregation thanks to the presence of hydrophilic (-SH) and hydrophobic (diphenyl rings) groups on the NP surface. The average diameters of AgNP and AgNP* calculated from a total of 30 particles were 55 ± 10 nm and 45 ± 10 nm, respectively.

**Figure 3:**
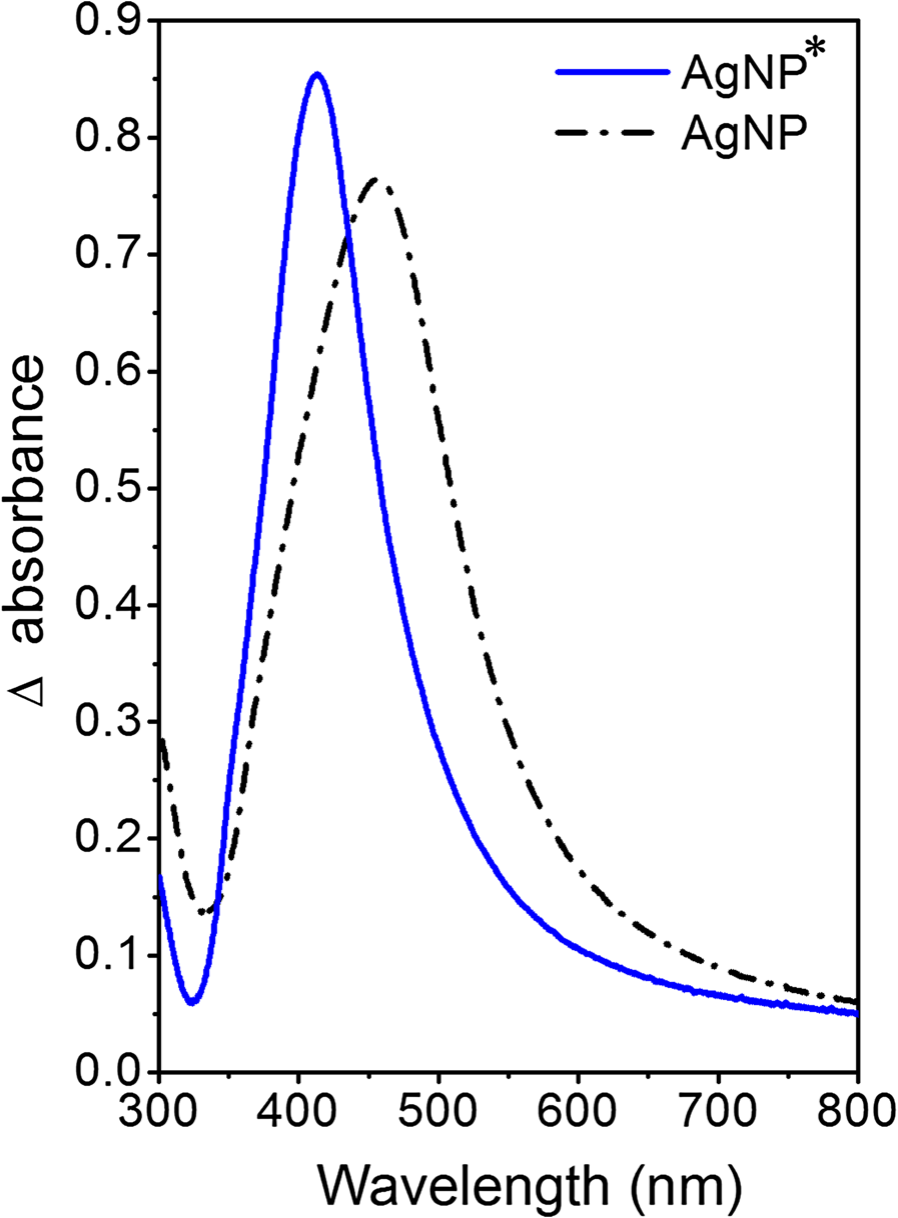
**UV-vis spectra of water solutions of silver nanoparticles and silver nanoparticles covered with dithiol.** Black scattered line = silver nanoparticles (AgNP); blue line = silver nanoparticles covered with dithiol (AgNP*).

The UV-vis absorption spectra of AgNP and AgNP* suspensions (Figure 3) showed well-defined plasmon bands at about 455 and 413 nm, which is characteristic of nanosized silver [18,19]. Absorption wavelength of surface plasmon resonance peak (SPR) is known to increase with nanoparticle size [20]. Observed wavelengths correspond well with average diameters of AgNPs estimated from TEM images (Figure 2A, B).

XPS analysis was used to monitor the change in the surface chemical composition after subsequent preparation steps. Atomic concentrations of C(1s), O(1s), S(2p), and Ag(3d) in pristine, plasma-modified PET and after grafting with BPD and silver nanoparticles are summarized in Table 1. After the plasma treatment, the PET surface is oxidized dramatically. Creation of oxygen-containing groups (carbonyl, carboxyl, hydroxyl, and ester) at the polymer surface is well known [21]. After grafting of plasma-treated PET with BPD, the oxygen concentration decreases dramatically. The attachment of BPD to the surface of PET (PET/BPD) was evidenced by the detection of sulfur with a concentration of 5.7 at.%. After next grafting with the AgNP and AgNP* particles, sulfur concentration decreased and silver is observed in the case of PET/plasma/BPD/AgNP samples, indicating AgNP presence on the sample surface. In the PET/plasma/AgNP* samples, the silver concentration is probably below the XPS detection limit. The presence of sulfur in this case, however, gives evidence of successful AgNP* attachment.

**Table 1:** Element concentrations of C, O, S, and Ag determined by XPS in surface polymer layer

**Sample**	**Element concentration (at.%)**			
	**C(1s)**	**O(1s)**	**S(2p)**	**Ag(3d)**
PET	72.5	27.5	-	-
PET/plasma	29.0	71.0	-	-
PET/plasma/BPD	75.4	18.9	5.7	-
PET/plasma/BPD/AgNP	75.0	23.1	1.1	0.8
PET/plasma/AgNP*	77.1	22.5	0.4	-

Pristine (PET), PET treated by plasma (PET/plasma), PET treated by plasma and grafted with BPD (PET/plasma/BPD), PET treated by plasma and grafted with BPD and then grafted with AgNP (PET/plasma/BPD/AgNP), and PET treated by plasma and grafted with AgNP* (PET/plasma/AgNP*) 4 days after the treatment.

**Figure 4:**
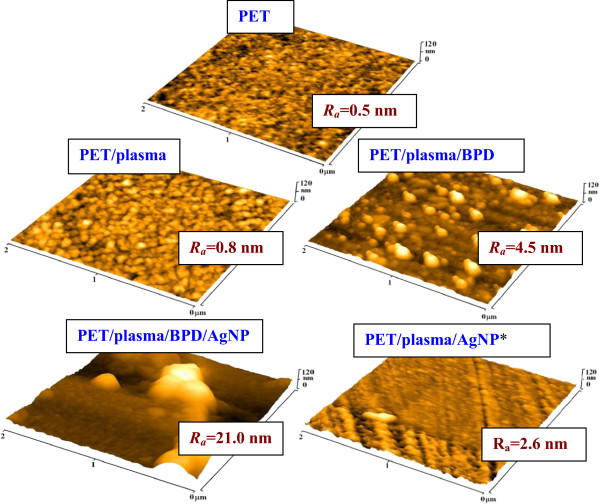
**AFM images.** AFM images of pristine PET (PET), PET treated by plasma and grafted with BPD (PET/plasma/BPD), PET treated by plasma and grafted with BPD and then with Ag nanoparticles (PET/plasma/BPD/AgNP), and PET treated by plasma and grafted with Ag nanoparticles previously grafted with dithiol (PET/plasma/AgNP*). *R*_a_ is surface roughness of samples in nanometers.

Surface morphology of PET treated by plasma and grafted with BPD and AgNP was studied by AFM method (Figure 4). Dramatic change in the surface morphology is observed after the plasma treatment and BPD grafting. After the plasma treatment and BPD grafting, the surface roughness of PET (*R*_a_ = 4.5 nm) is significantly higher than that of plasma-treated PET (*R*_a_ = 0.8 nm). Another dramatic increase in surface roughness is observed after attachment of AgNPs (*R*_a_ = 21.0 nm). It is evident that a significant aggregation of AgNPs takes place during particle grafting. It could be caused by the surface energy of plasma-treated PET. In the case of AgNP* grafted samples, the surface morphology resembles that of the PET/plasma/BPD sample but with a lower concentration of BPD molecules. This finding is in accord with the XPS results described above.

**Figure 5:**
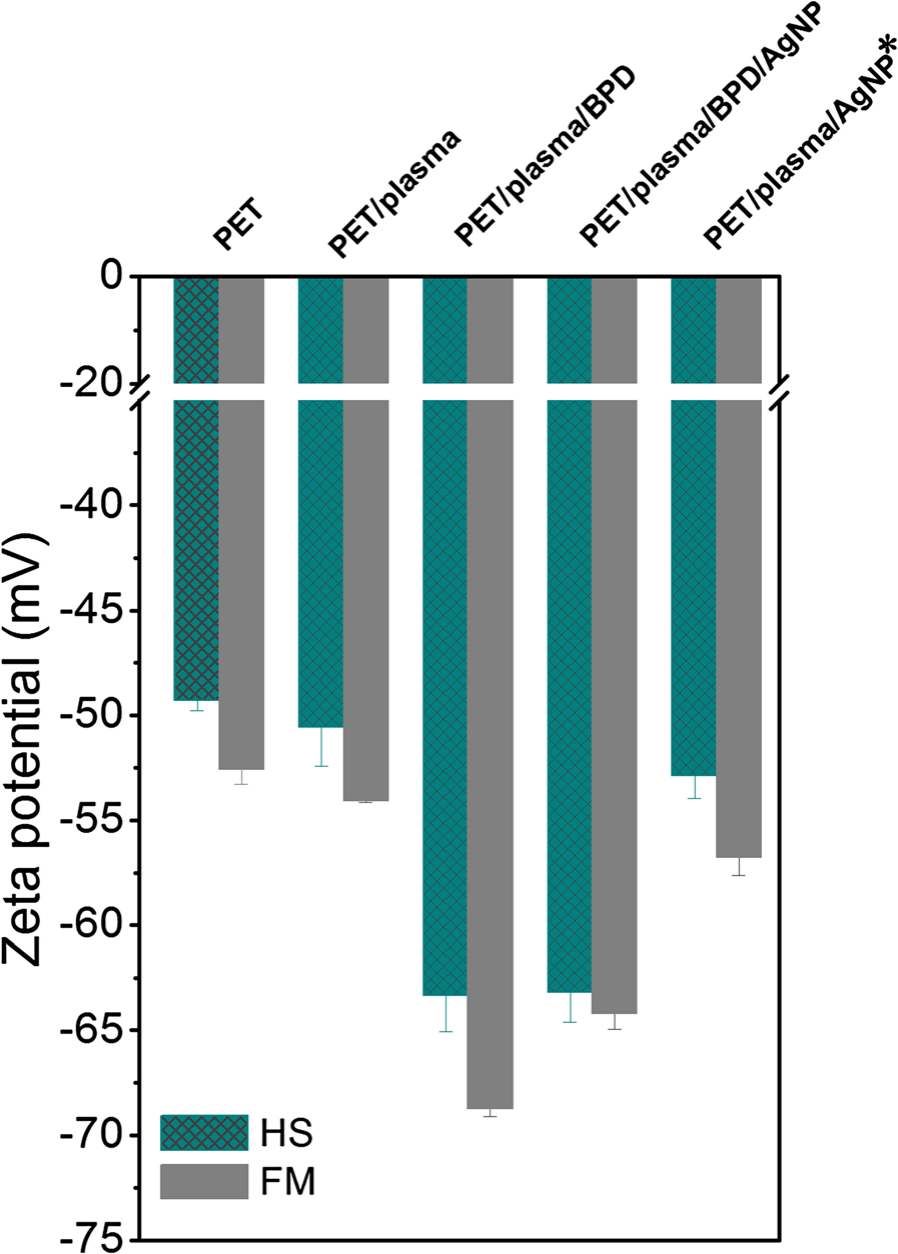
**Zeta potential.** Zeta potential determined on pristine (PET), PET treated by plasma (PET/plasma), PET treated by plasma and grafted with BPD (PET/plasma/BPD), PET treated by plasma and grafted with BPD and then subsequently with Ag nanoparticles (PET/plasma/BPD/AgNP), and PET treated by plasma and grafted with Ag nanoparticles previously grafted with dithiol (PET/plasma/AgNP*). HS means data obtained by the streaming current method and Helmholtz-Smoluchowski equation; FM means data obtained by the streaming potential method and Fairbrother-Mastins equation.

Similar results were obtained by electrokinetic analysis (Figure 5). After BPD grafting of plasma-treated PET, zeta potential decreases in comparison with pristine PET due to the presence of -SH groups and diphenyl rings of dithiol on the sample surface. Another change of surface chemistry and charge is visible after the grafting with AgNPs, which is due to the presence of AgNPs on the sample surface. Since the silver concentration is low, the observed change is low, too. Grafting of the plasma-treated PET with AgNP* particles leads to only negligible change in zeta potential (compare PET/plasma and PET/plasma/AgNP* cases in Figure 5). Small change in zeta potential shows that only a small amount of AgNP* particles is attached in this case. All these findings are in accord with the results of XPS analysis described above (see also Table 1).

The systems studied may have potential application, e.g., in medicine as prevention of creation of bacterial biofilm [22].

## Conclusions

Two different procedures were used for coating of PET surface with silver nanoparticles. Both procedures are based on the surface activation of PET by Ar plasma discharge and use of dithiol as binding reagent between silver nanoparticles and plasma-modified PET surface. XPS results confirmed creation of a silver nanoparticle-thiol layer (in the case of AgNP) on the PET surface. Rather large objects observed on AFM images show that a significant aggregation of deposited AgNPs takes place during the grafting procedure. Grafting with thiols and gold nano-objects generally leads to a decrease of the zeta potential. We achieved higher concentration of silver nanoparticles by deposition on PET grafted beforehand with dithiol.

## Competing interests

The authors declare that they have no competing interests.

## Authors’ contributions

AR carried out the AFM analysis, evaluated the surface morphology and roughness, and wrote and designed the study. ZN analyzed the chemical and optical properties of AgNPs and silver-grafted PET. ZK performed zeta potential measurement. VS participated in the study coordination and paper correction. All authors read and approved the final manuscript.

## References

[B1] Gam-Derouich S Mahouche-Chergui S Truong S Ben Hassen-Chehimi D Chehimi MM Design of molecularly imprinted polymer grafts with embedded gold nanoparticles through the interfacial chemistry of aryl diazonium salts Polymer 2011 9 4463 4470 10.1016/j.polymer.2011.08.007

[B2] Guerrouache M Mahouche-Chergui S Chehimi MM Carbonnier B Site-specific immobilisation of gold nanoparticles on a porous monolith surface by using a thiol–yne click photopatterning approach Chem Commun 2012 9 7486 7488 10.1039/c2cc33134a 22728408

[B3] Sharma VK Yngard RA Lin Y Silver nanoparticles: green synthesis and their antimicrobial activities Adv Colloid Interfac 2009 9 83 89 10.1016/j.cis.2008.09.002 18945421

[B4] Krutyakov YA Kudrynskiy AA Olenin AY Lisichkin GV Synthesis and properties of silver nanoparticles: advances and prospects Russ Chem Rev 2008 9 233 257 10.1070/RC2008v077n03ABEH003751

[B5] Monteiro DR Gorup LF Takamiya AS Ruvollo AC Camargo ER Barbosa DB The growing importance of materials that prevent microbial adhesion: antimicrobial effect of medical devices containing silver Int J Antimicrob Agents 2009 9 103 110 10.1016/j.ijantimicag.2009.01.017 19339161

[B6] Ahamed M AlSalhi MS Siddiqui MKJ Silver nanoparticle applications and human health Clin Chim Acta 2010 9 1841 1848 10.1016/j.cca.2010.08.016 20719239

[B7] García-Barrasa J López-de-luzuriaga JM Monge M Silver nanoparticles: synthesis through chemical methods in solution and biomedical applications Cent Eur J Chem 2011 9 7 19 10.2478/s11532-010-0124-x

[B8] Tran QH Nguyen VQ Le AT Silver nanoparticles: synthesis, properties, toxicology, applications and perspectives Adv Nat Sci: Nanosci Nanotechnol 2013 9 033001 10.1088/2043-6262/4/3/033001

[B9] Omastova M Mičušík M Polypyrrole coating of inorganic and organic materials by chemical oxidative polymerization Chem Pap 2012 9 392 414 10.2478/s11696-011-0120-4

[B10] Li C Bai H Shi GQ Conducting polymer nanomaterials: electrosynthesis and applications Chem Soc Rev 2009 9 2397 2409 10.1039/b816681c 19623357

[B11] Yagci Y Jockusch S Turro NJ Photoinitiated polymerization: advances, challenges, and opportunities Macromolecules 2010 9 6245 6260 10.1021/ma1007545

[B12] Mahouche-Chergui S Guerrouache M Carbonnier B Chehimi MM Polymer-immobilized nanoparticles Colloid Surf A 2013 9 43 68

[B13] Řezníčková A Kolská Z Hnatowicz V Stopka P Švorčík V Comparison of argon plasma-induced surface changes of thermoplastic polymers Nucl Instrum Meth B 2011 9 83 88 10.1016/j.nimb.2010.11.018

[B14] Smith SL Nissamudeen KM Philip D Gopchandran KG Studies on surface plasmon resonance and photoluminescence of silver nanoparticles Spectrochim Acta A 2008 9 186 190 10.1016/j.saa.2007.12.002 18222106

[B15] Řezníčková A Kolská Z Siegel J Švorčík V Grafting of gold nanoparticles and nanorods on plasma-treated polymers by thiols J Mater Sci 2012 9 6297 6304 10.1007/s10853-012-6550-8

[B16] Lu M Li XH Yu BZ Li HL Electrochemical behavior of Au colloidal electrode through layer-by-layer self-assembly J Colloid Interf Sci 2002 9 376 382 10.1006/jcis.2002.8238 16290541

[B17] Kolská Z Řezníčková A Švorčík V Surface characterization of polymer foilse-polymers 2012 9 1 6

[B18] Yin J Yang Y Hu ZQ Deng BL Attachment of silver nanoparticles (AgNPs) onto thin-film composite (TFC) membranes through covalent bonding to reduce membrane biofouling J Membrane Sci 2013 9 73 82

[B19] Kim JS Kuk E Yu KN Kim JH Park SJ Lee HJ Kim SH Park YK Park YH Hwang CY Kim YK Lee YS Jeong DH Cho MH Antimicrobial effects of silver nanoparticles Nanomed-Nanotechnol 2007 9 95 101 10.1016/j.nano.2006.12.001 17379174

[B20] Mayoral A Barron H Estrada-Salas R Vazquez-Duran A Jose-Yacamán M Nanoparticle stability from the nano to the meso interval Nanoscale 2010 9 335 342 10.1039/b9nr00287a 20644815

[B21] Chu PK Chen JY Wang LP Huang N Plasma-surface modification of biomaterials Mater Sci Eng R 2002 9 143 206 10.1016/S0927-796X(02)00004-9

[B22] Webb HK Crawford RJ Sawabe T Ivanova EP The systems studied may have potential application e.g. in medicine as prevention of creation of bacterial biofilm Microbs Environ 2009 9 39 42 10.1264/jsme2.ME08538

